# Correlation of in vitro genotoxicity and oncogenicity induced by radiation and asbestos fibres.

**DOI:** 10.1038/bjc.1985.231

**Published:** 1985-10

**Authors:** T. K. Hei, C. R. Geard, R. S. Osmak, M. Travisano

## Abstract

The in vitro cytotoxicity and oncogenic potential of both native and acid leached asbestos fibres were studied using the C3H 10T1/2 cell model. Both native and leached fibres induced a dose-dependent toxicity. At high fibre concentrations, acid leached fibres were significantly less toxic than their untreated counterparts. While asbestos fibres alone do not induce oncogenic transformation at the concentration examined, it was found that both leached and native fibres substantially enhanced the oncogenicity of gamma-irradiation in a more than additive fashion. Although no significant chromosomal aberrations or sister chromatid exchanges (SCE) were found in asbestos treated cultures, a significantly higher number of SCEs was observed in cells treated with both asbestos and radiation compared to cells receiving radiation alone. The results suggest that the enhancement in radiation induced oncogenicity by asbestos fibres may be attributed to the mere physical presence of the fibres rather than any chemical contaminants the fibres may contain. Furthermore, the carcinogenicity of asbestos may be unrelated to genotoxicity.


*Br. J. Cancer* (1985), **52**, 591–597

### Correlation of *in vitro* genotoxicity and oncogenicity induced by radiation and asbestos fibres

T.K. Hei, C.R. Geard, R.S. Osmak & M. Travisano

*Radiological Research Laboratory, College of Physicians & Surgeons of Columbia University, New York, NY 10032, USA.*

**Summary** The *in vitro* cytotoxicity and oncogenic potential of both native and acid leached asbestos fibres were studied using the $C_3H$ 10T1/2 cell model. Both native and leached fibres induced a dose-dependent toxicity. At high fibre concentrations, acid leached fibres were significantly less toxic than their untreated counterparts. While asbestos fibres alone do not induce oncogenic transformation at the concentration examined, it was found that both leached and native fibres substantially enhanced the oncogenicity of gamma-irradiation in a more than additive fashion. Although no significant chromosomal aberrations or sister chromatid exchanges (SCE) were found in asbestos treated cultures, a significantly higher number of SCEs was observed in cells treated with both asbestos and radiation compared to cells receiving radiation alone. The results suggest that the enhancement in radiation induced oncogenicity by asbestos fibres may be attributed to the mere physical presence of the fibres rather than any chemical contaminants the fibres may contain. Furthermore, the carcinogenicity of asbestos may be unrelated to genotoxicity.

Asbestos has been shown to be carcinogenic to both man (Barum & Truan, 1958; Wagner *et al.*, 1960; Selikoff *et al.*, 1979) and experimental animals (Wagner *et al.*, 1973; Gross *et al.*, 1967). Two specific types of malignancy are associated with human exposure to asbestos: primary bronchogenic carcinoma of the lung and diffuse mesothelioma of the pleura and peritoneum (Hasan *et al.*, 1978). The mechanism by which asbestos produces malignancy is not known. It has been shown that the mere physical presence of the asbestos fibres can induce tumours in animals. This carcinogenicity does not appear to be related to any contaminants of native fibres such as various hydrocarbons or trace metals (Wagner & Berry, 1969). Fibres that are long and thin are especially carcinogenic when tested in experimental animals compared with those that are short and thin (Stanton *et al.*, 1977).

*In vitro* studies in tissue culture have shown that asbestos fibres tend to be phagocytosed by a variety of cell types, including macrophages (McLemore *et al.*, 1979; Miller *et al.*, 1978), V79-4 lung cells (Huang *et al.*, 1979) and mouse embryo cells (Dourmashkin & Dougherty, 1961). Small fibres tend to be completely phagocytosed while longer fibres may be only partially engulfed. The perturbation of plasma membranes together with the presence of various polycycloc aromatic hydrocarbons such as benzo(a)pyrene in cigarette smoke could partially explain the high incidence of lung cancers observed in asbestos workers (Selikoff *et al.*, 1968) and the transformation frequency observed with cells in culture (Brown *et al.*, 1983; Hesterberg & Barrett, 1984).

Recent studies have shown that asbestos fibres can also potentiate the *in vitro* oncogenicity of radiation (Hei *et al.*, 1984*a*). In an attempt to clarify whether such potentiation could be due to chemical contaminants the native fibres may contain, the ability of acid-leached asbestos fibres to influence radiation induced transformation incidence in $C_3H$ 10T1/2 cells was investigated. Furthermore, in order to examine a possible mechanistic basis for asbestos potentiation of radiation induced transformation at the chromosomal level and to evaluate genotoxicity of the various treatments, cytokinetics, chromosomal aberrations and sister chromatid exchanges were assessed after exposure of $C_3H$ 10T1/2 cells to asbestos fibres, either alone or in conjunction with radiation.

### Materials and methods

$C_3H$ 10T1/2 mouse embryo fibroblasts were used for these studies. The fibre preparation and culture conditions have been described previously (Hei *et al.*, 1984*a*). Basically, UICC standard reference samples of crocidolite and amosite were weighed out, suspended in distilled water, sterilized by autoclaving and used at the concentrations indicated. For those experiments where the influence of possible chemical contaminants of native UICC fibres was to be determined, samples of crocidolite and amosite ($\sim$100 mg) were suspended in 30 ml of 5 N HCl. The samples were stirred thoroughly to disperse the fibres and leached

Correspondence: T.K. Hei
Received 29 April 1985; and in revised form, 22 May 1985

© The Macmillan Press Ltd., 1985

for 48 h at room temperature. Following leaching, the fibres were rinsed in distilled water twice by centrifugation. The fibres were then weighed, suspended in distilled water and sterilized as before.

*In vitro* cytotoxicity, growth rate, and oncogenicity of native and leached UICC asbestos fibres on $C_3H$ 10T1/2 were determined. The details have been described previously (Hei *et al.*, 1984*a*). Briefly, various numbers of $C_3H$ 10T1/2 cells in exponential-phase of growth were plated per 100 mm diameter petri dishes such that after treatment, 50 to 60 viable clones would survive. Asbestos fibres, suspended in 10 ml complete medium were added to the cultures 18–24 h later in concentrations ranging from 2.5–50 $\mu g\,ml^{-1}$. Cells treated with fibres for 24 h were washed twice with buffered salt solution, replenished with fresh media and stained after 10–12 days incubation.

Growth rate and saturation density of asbestos treated cultures were determined by plating $5 \times 10^4$ cells per dish and treated with fibres for 24 h at the concentrations described above. At each time point studied, triplicate dishes from each treatment group were trypsinized and cells counted separately using a Coulter counter.

For the transformation assay, exponentially growing cells were plated in 100 mm diameter petri dishes at a density such that ~300–400 viable cells would survive a 24 h asbestos pretreatment at a concentration of 5 $\mu g\,ml^{-1}$, or a 4 Gy dose of gamma irradiation, or a combination of both. The source of gamma rays was a Cesium-137 irradiator, and the absorbed dose rate was 1.36 Gy $min^{-1}$. The treated cells were then washed, replenished with fresh medium and incubated for 6 weeks with medium changed every 10 days. The cultures were then fixed, stained and type II and III foci scored as transformants (Reznikoff *et al.*, 1973) as described previously (Hei *et al.*, 1984*a*).

Asbestos-treated and/or gamma-irradiated cells were incubated with bromodeoxyuridine (BrdU, $3 \times 10^{-6}$ M) as a monitor of cell cycle progression and for SCE studies, while sequential 3 or 4 h colcemid ($5 \times 10^{-7}$ M) treatments were used to accumulate mitotic cells (Hei *et al.*, 1984*b*) after irradiation. Cells were fixed at various times from 4–120 h after initiation of asbestos treatment and stained with 2% Giemsa in 0.3 M $Na_2HPO_4$, pH 10.4 Mitotic indices (1–2000 cells per point), frequencies of mitoses that had passed through 1,2 or 3 replication (400 cells per point) and SCEs per chromosome (1000 from second mitotic division cells) were then scored on coded slides.

Data were analyzed using the two-tailed Student's *t* test for unpaired data. Differences between means were regarded as significant if $P < 0.05$.

### Results

As reported previously (Hei *et al.*, 1984*a*), both crocidolite and amosite asbestos fibres were found to be cytotoxic to $C_3H$ 10T1/2 cells in a dose-dependent manner. Leaching the fibres with 5N HCl for 48 h did not alter the toxicity of fibres at lower dose ranges (<20 $\mu g\,ml^{-1}$, Figure 1). At higher fibre concentrations, leached fibres were substantially less toxic than their plain or untreated counterparts. A similar cytotoxic response was found with amosite fibres (data not shown). This insensitivity, however, was only observed in <5% of the exposed cell populations and may reflect the reduced ability of a small fractions of cells to take up washed fibres. A concentration of 5 $\mu g\,ml^{-1}$ of both crocidolite and amosite fibres was chosen for all other studies since ~60% of cells possess clonogenic potential after 24 h fibre treatments. This concentration was therefore representative of a low toxicity protocol, desirable in carcinogenicity studies.

**Figure 1** Effects of plain or acid leached crocidolite fibres on surviving fractions of $C_3H$ 10T1/2 cells. Cells were treated with various concentrations of fibres for 24 h, washed and the number of colonies formed per dish was counted after 10–12 days. Results are pooled data from two experiments. Four dishes per concentration were used in each study. (●, plain; ▲, acid-leached).

Figure 2 shows the effects of either plain or acid leached amosite asbestos fibres on the growth kinetics of $C_3H$ 10T1/2 cells after a 24 h treatment period (results for crocidolite fibres were similar and are not shown). At both fibre concentrations the unwashed fibres produced a greater initial delay in growth yet once this delay was overcome the slopes of the growth curves were very similar. This applies at both $5\,\mu g\,ml^{-1}$ (plain *vs.* leached) and $25\,\mu g\,ml^{-1}$ (plain *vs.* leached). It is possible that there was some factor(s) on plain fibres which, while not cytotoxic, acted initially to delay but not inhibit cellular growth. Since leached fibres act differently from plain fibres to some extent, it is highly pertinent to consider whether the incidence of transformation is concomitantly affected. Such a comparison is presented in Table I. The plating efficiencies of $C_3H$ 10T1/2 cells in two separate experiments were 26 and 32% respectively, while a dose of 4 Gy of $^{137}Cs$ gamma rays reduced the percentage of surviving cells to ~38%. Combined treatments of asbestos fibres (plain or leached) and gamma rays results in a percentage of cells surviving which was compatible with an additive effect of the two modalities and with no difference between plain or leached fibres. Clearly, combined treatments have a greater than additive effect on the incidence of oncogenic transformation by a factor of 2–3. For both crocidolite and amosite treatments the frequency of transformation was increased by 50–60% over control but this increase was not statistically significant. After 4 Gy, transformation incidence was increased by ~300% (highly statistically significant $P<0.01$) while the combined modalities increased transformation frequencies by ~1,000% over control level. In no instance was there any indication that the status of the fibres influenced their potentiation of the response to radiation. Clearly then the cocarcinogenic effect of the asbestos fibres for radiation-induced transformation cannot be attributed to leachable contaminants, and must reflect a property of the fibres themselves.

The effects of asbestos and/or radiation on cellular progression through the cell cycle are shown in Figure 3 and 4. Mitotic cells were sequentially accumulated with colcemid at 4 h intervals from 0–20 h post irradiation (4 Gy $^{137}Cs$ gamma rays) which was given after a 24 h treatment of cells with plain asbestos fibres ($5\,\mu g\,ml^{-1}$). A straight line relationship on a cumulative mitotic index polot *versus* time is indicative of a constant rate of flow of cells into mitosis, while the slope represents the rate of flow. The data indicate that in the presence of asbestos fibres fewer cells were cycling than controls. A dose of 4 Gy induced a substantial delay (~8 h) in the mitotic cycle while

**Figure 2** Effects of plain or acid leached amosite fibres on growth rate of $C_3H$ 10T1/2 cells. Each point represents averages of two experiments. (●, control untreated; ▲, $5\,\mu g\,ml^{-1}$ plain; ▼, $5\,\mu g\,ml^{-1}$ leached; ■, $25\,\mu g\,ml^{-1}$ plain; ◆, $25\,\mu g\,ml^{-1}$ leached).

the combined fiber-radiation treatments resulted in no consistent extra discernible increase in induced delay. Using BrdU as a monitor of cell cycle progression, the frequencies of mitoses post-treatment were followed. After the radiation treatments there were too few mitoses in the 0 to 4 and 4 to 8 h collection periods to accurately determine cell cycle status (Figure 4). The histograms show however that asbestos fibres (upper panels, Figure 4) have little effect on cycling cells. At 28 h after initiation of fibre treatment ~90% of mitoses have passed through two DNA synthesis periods (2nd division mitoses) while at 48 h ~90% have passed through three DNA synthesis periods. The curves represent fits through the midpoints of the collection periods and show that for the control and both asbestos fiber treatments ~50% of mitoses are 3rd divisions 37 h after beginning BrdU incorporation. After 4 Gy of gamma rays this time is increased by ~2 h, with a further 2 h increment for the crocidolite/gamma ray group. The time to 50% 3rd mitotic divisions, however, is extended to 45 h in the amosite/gamma-

**Table I** Effects of gamma-irradiation and leached or native UICC asbestos fibres on transformation incidence of $C_3H$ 10T1/2 cells.

| *Treatment* | *SF* | *Total cells*[a] *at risk* $n \times 10^4$ | *No. of transformed foci* *Type II* | *Type III* | *TF* ($\times 10^{-4}$) | |
|---|---|---|---|---|---|---|
| 4 Gy $\gamma$ rays | 0.36 | 2.94 | 3 | 0 | 1.02 | |
| | | | | | | 1.4 |
| | 0.40 | 1.18 | 0 | 2 | 1.69 | |
| Crocidolite/$\gamma$ rays | | | | | | |
| 5 $\mu$g ml$^{-1}$ plain | 0.11 | 2.04 | 2 | 9 | 5.50 | |
| | | | | | | 4.9 |
| 5 $\mu$g ml$^{-1}$ leached | 0.18 | 2.13 | 1 | 8 | 4.30 | |
| Amosite/$\gamma$ rays | | | | | | |
| 5 $\mu$g ml$^{-1}$ plain | 0.20 | 1.10 | 1 | 3 | 3.66 | |
| | | | | | | 4.8 |
| 5 $\mu$g ml$^{-1}$ leached | 0.22 | 1.03 | 4 | 2 | 5.90 | |
| Control | (0.26) PE | 1.98 | 0 | 1 | 0.51 | |
| | | | | | | 0.46 |
| | (0.32) | 4.03 | 1 | 1 | 0.40 | |
| Crocidolite | | | | | | |
| 5 $\mu$g ml$^{-1}$ plain | 0.57 | 4.30 | 1 | 2 | 0.70 | |
| | | | | | | 0.79 |
| 5 $\mu$g ml$^{-1}$ leached | 0.54 | 3.42 | 2 | 1 | 0.88 | |
| Amosite | | | | | | |
| 5 $\mu$g ml$^{-1}$ plain | 0.65 | 1.17 | 1 | 0 | 0.86 | |
| | | | | | | 0.75 |
| 5 $\mu$g ml$^{-1}$ leached | 0.49 | 1.57 | 1 | 0 | 0.64 | |

SF = Surviving fraction; PE = Plating efficiency; TF = Transformation frequencies (transformants per surviving cell). [a] = Average 300 to 400 viable cells per dish.

**Figure 3** Percent cumulative mitotic index as a function of time after irradiation in $C_3H$ 10T1/2 cells. Cells were pretreated with 5 $\mu$g ml$^{-1}$ asbestos fibres for 24 h, irradiated, washed and replenished with fresh medium. Sample dishes from various treatment groups were processed every 4 h to determine the mitotic index. (▲, Control; ◆, crocodilite; ▼, amosite; ●, $\gamma$ rays; ■, $\gamma$ rays + crocodilite; ×, $\gamma$ rays + amosite).

ray treated cells, a 9 h increase over that due to amosite alone. In terms of cellular kinetics, a 24 h treatment of $C_3H$ 10T1/2 cells with plain asbestos fibres results in a reduction in the fraction of cycling cells but has little effect on the cell cycle times of those cells that are cycling. After 4 Gy of gamma radiation either alone or in conjunction with asbestos, the number of cells in mitosis decline dramatically for 8 h, primarily reflecting an effect on cells in $G_2$. The asbestos fibres, particularly amosite, then have a further enhancing effect on induced delay.

When mitoses were examined for chromosomal aberrations after treatment, over the five collection periods (up to 24 h) there were $0.04 \pm 0.016$ aberrations per cell for controls, $0.16 \pm 0.045$ aberrations per cell for the 24 h plain crocidolite fibre treatment and $0.09 \pm 0.019$ aberrations per cell for the plain amosite fibre treatment. These increases are significantly different from the control ($P < 0.05$) showing that asbestos fibres are inefficient inducers of chromosomal aberrations. However, these levels of aberrations certainly cannot explain the asbestos cytotoxicity since 24 h fibre treatments results in 60 to 70% cell survival relative to controls (Hei *et al.*, 1984*a*; Figure 1, Table I), yet 90 and 92% of mitoses are aberration-free after crocidolite and amosite treatments respectively compared to 97% for controls (upper

**Figure 4** Percent 1st division mitoses (bottom histograms) and 3rd division mitoses (upper histograms) in $C_3H$ 10T1/2 cells after asbestos ($5\,\mu g\,ml^{-1}$) or radiation (4 Gy) treatment or a combination of both. Ordinate indicates time after either a 24 h fiber treatment or fibres plus radiation exposure.

panel of Figure 5). Figure 5 also shows the frequencies of aberrations per cell after radiation and asbestos treatments. Over the 6 collection periods 4 Gy alone produced a mean of 3.3 aberrations per cell, yet 21% of cells were aberration-free, while 4 Gy plus crocidolite produced 2.6 aberrations per cell with 26% of cells aberration-free, and 4 Gy plus amosite 3.0 aberrations per cell and 19% of cells aberration-free. Clearly some cells sustain very high levels of induced damage while a similar minority are undamaged, however there is no increase in aberrations when asbestos is combined with radiation nor is there a difference in the spectrum of aberrations observed (Figure 5 deletions *versus* interchanges).

It is not possible then to explain the enhanced effect of asbestos fibres on radiation-induced transformations in terms of increased frequencies of chromosomal aberrations, particularly chromosomal interchanges.

The frequencies of sister chromatid exchanges (SCEs) per chromosome from 2nd division mitoses only and from all treatments are shown in Figure 6. Frequencies were consistent and similar at ~0.3 SCEs per chromosome and not significantly

**Figure 5** Chromosomal aberrations per $C_3H$ 10T1/2 cells after the various treatments. Results are means from 6 collection periods; up to 24 h post-treatment. Upper histograms are results expressed as percent of cells without any aberrration.

**Figure 6** Sister chromatid exchanges per chromosome in $C_3H$ 10T1/2 cells after either asbestos or radiation treatment or a combination of both as a function of time post-treatment. (○, Control; □, Crocodilite; △, amosite; ●, 4 Gy; ■, 4 Gy + crocodilite; ▲, 4 Gy + amosite).

different for the plain asbestos treatments relative to control. After 4 Gy gamma-irradiation, either alone or in conjunction with asbestos, a pronounced increase in SCE was seen. At the 5 periods assessed after irradiation, radiation plus asbestos produced more SCEs per chromosome than radiation alone. (At the 0–4 h post treatment period there were too few mitoses.) Overall 4 Gy alone resulted in $0.46 \pm 0.016$ SCEs per chromosome (0.165 induced SCEs per chromosome) which is significantly different ($P < 0.01$) from $0.52 \pm 0.018$ and $0.56 \pm 0.02$ SCEs per chromosome for 4 Gy plus amosite and 4 Gy plus crocidolite, respectively i.e. the presence of asbestos fibres enhances the likelihood of radiation inducing SCEs and this result is similar to, though certainly less pronounced than, that seen for asbestos potentiated oncogenic transformation.

### Discussion

The fact that asbestos is carcinogenic and has been extensively used by industries and households makes it an important health concern. It is not known whether a threshold concentration of asbestos is involved in the carcinogenic process.

The carcinogenic mechanism of asbestos is not known. Previous epidemiological data have demonstrated a synergism between asbestos fibres and cigarette smoke on lung cancer incidence among cigarette smoking workers (Selikoff *et al.*, 1968). Similar cocarcinogenic effects of asbestos have subsequently been shown with polycyclic aromatic hydrocarbons in both rats (Salk & Vosamae, 1975) and hamsters (Miller *et al.*, 1965). Recent *in vitro* studies have also found a potentiating effect of benzo(a)pyrene and radiation induced oncogenic transformation incidence (DiPaolo *et al.*, 1983; Brown *et al.*, 1983; Hei *et al.*, 1984*a*).

The present findings that either native or acid-leached asbestos fibres can potentiate the oncogenicity of radiation suggested that the mere physical presence of the fibres is responsible. Although asbestos has been shown to increase cellular uptake while decreasing the metabolism of benzo(a)pryene and can partially explain the high oncogenicity of the two together (Eastman *et al.*, 1983), such is not the case with radiation.

Previous studies by this laboratory have demonstrated a dose response cytotoxicity of $C_3H$ 10T1/2 cells exposed for 24 h to graded doses of asbestos fibres (Hei *et al.*, 1984*a*). To determine whether the toxicity may be contributed by an extractable moiety bound to asbestos, the fibres were leached with hydrocholoric acid which has been demonstrated previously capable of removing $Ca^{++}$ and $Mg^{++}$ ions from the fibres (Reiss *et al.*, 1980). The decrease in toxicity of leached fibres at higher doses suggested that surface metal ions may contribute to the observed toxicity.

The present findings indicate that asbestos, at concentrations which alone are ineffective for the induction of oncogenic transformation *in vitro* and yet potentiate the oncogenicity of gamma rays, do not appreciably affect the cell cycle kinetics assessed by both BrdU incorporation and cellular growth curve. Although no significant increases in SCEs were found in asbestos treated cultures, a significantly higher number of SCEs was observed in cells treated with both asbestos and radiation compared to cells receiving radiation alone. Several previous studies have shown that asbestos is either negative in including SCEs above control levels (Kaplan *et al.*, 1980) or is only marginally active at high concentrations (Oshimura *et al.*, 1984). The facts that asbestos produces no DNA strand break in mammalian cells (Mossman *et al.*, 1983) nor back mutations in bacteria in the presence of a metabolic activation system (Chamberlain *et al.*, 1977) suggest that the carcinogenicity of asbestos is unrelated to genotoxicity.

This work was supported in part by grant CA 12536-13 of the National Cancer Institute, National Institute of Health.

### References

BARUM, D.C. & TRUAN, T.D. (1958). An epidemiological study of lung cancer in asbestos miners. *A.M.A. Arch. Indust. Hyg. Occup. Med.*, **17**, 634.

BROWN, R.C., POOLE, A. & FLEMING, G.T.A. (1983). The influence of asbestos dust on the oncogenic transformation of $C_3H$ 10T1/2 cells. *Cancer Lett.*, **18**, 221.

CHAMBERLAIN, M. & TARMY, E.M. (1977). Asbestos and glass fibers in bacterial mutation tests. *Mutation Res.*, **43**, 159.

DIPAOLO, J.A., DEMARINIS & DONIGER, J. (1983). Asbestos and benzo-(a)pyrene synergism in the transformation of Syrian hamster embryo cells. *Pharmacology*, **27**, 65.

DOURMASHKIN, R.R. & DOUGHERTY, R.M. (1961). Phagocytosis of chrystalline particles by cells grown in tissue culture. *Exp. Cell Res.*, **25**, 400.

EASTMAN, A., MOSSMAN, B.T. & BRESNICK, E. (1983). Influence of asbestos on the uptake of benzo(a)pyrene and DNA alkylation in hamster tracheal epithelial cells. *Cancer Res.*, **43**, 1251.

GROSS, P., DcTREDELLE, R.T.P., TOLKER, E., KASCHAK, M. & BABYAK, M.A. (1967). Experimental asbestosis: The development of lung cancer in rats with pulmonary deposits of chrysotile asbestos dust. *Arch. Environ. Health*, **15**, 343.

HASAN, F.M., NASH, G. & KAZEMI, H. (1978). Asbestos exposure and related neoplasia, the 28 year experience of a major urban hospital. *Am. J. Med.*, **65**, 649.

HEI, T.K., HALL, E.J. & OSMAK, R.S. (1984*a*). Asbestos, radiation and oncogenic transformation. *Br. J. Cancer*, **50**, 717.

HEI, T.K., GEARD, C.R. & HALL, E.J. (1984*b*). Effects of cellular non-protein sulfhydryl depletion in radiation induced oncogenic transformation and genotoxicity in mouse $C_3H$ 10T1/2 cells. *Int. J. Radiation Oncology Biol. Phys.*, **10**, 1255.

HESTERBERG, T. & BARRETT, J.C. (1984). Dependence of asbestos and mineral dust-induced transformation of mammalian cells in culture on fiber dimension. *Cancer Res.* **44**, 2170.

HUANG, S.L. (1979). Amosite, chrysotile and crocidolite asbestos are mutagenic in Chinese hamster lung cells. *Mutation Res.*, **68**, 265.

KAPLAN, H., RENIER, A., JAURAND, M.C. & BIGNON, R. (1980). Sister chromatid exchanges in mesothelial cells cultured with chrysotile asbestos. In *The In Vitro Effects of Mineral Dusts*, p. 251 (Ed. Brown *et al.*) Academic Press: New York.

MCLEMORE, T., CORSON, M., MACE, M. & 6 others. (1979). Phagocytosis of asbestos fibers by human pulmonary alveolar macrophasges. *Cancer Lett.*, **6**, 183.

MILLER, K., HANDFIELD, R.I.M. & KAGAN, E. (1978). The effects of different mineral dusts on the mechanism of phagocytosis: A scanning electron microscope study. *Environ. Res.*, **15**, 139.

MILLER, L., SMITH, W.E. & BERLINER, S.W. (1965). Tests for effect of asbestos on benzo(a)pyrene carcinogenesis in the respiratory tract. *Ann. NY Acad. Sci.*, **132**, 489.

MOSSMAN, B.T., EASTMAN, A., LANDESMAN, J.M. & BRESNICK, E. (1983). Effects of crocidolite and chrysotile asbestos on cellular uptake and metabolism of benzo(a)pyrene in hamster tracheal epithelial cells. *Environ. Health Perspect.*, **51**, 331.

OSHIMURA, M., HESTERBERG, T.W., TSUTSIU, T. & BARRETT, J.C. (1984). Correlation of asbestos induced cytogenetic effects with cell transformation of Syrian hamster embryo cells in culture. *Cancer Res.*, **44**, 5107.

REISS, B., SOLOMON, S., WEISBURGER, J.H. & WILLIAMS, G.M. (1980). Comparative toxicities of different forms of asbestos in a cell culture assay. *Environ. Res.*, **22**, 109.

REZNIKOFF, C.A., BERTRAM, J.S., BRANKOW, D.W. & HEIDELBERGER, C. (1973). Quantitative and qualitative studies of chemical transformation of clonal $C_3H$ mouse embryo cells sensitive to post-confluence inhibition of cell division. *Cancer Res.*, **33**, 3239.

SALK, R. & VOSAMAE, A. (1975). Induction of lung tumors in rats by intratracheal instillation of benzo(a)-pyrene and chrysotile asbestos dust. *Exp. Clin. Oncology*, **2**, 88.

SELIKOFF, I.J., HAMMOND, E.C. & CHUNG, J. (1968). Asbestos exposure, smoking and neoplasia. *JAMA*, **204**, 106.

SELKOFF, I.J., HAMMOND, E.C. & SEIDMAN, H. (1979). Mortality experience of insulation workers in the U.S. and Canada. *Ann. NY Acad. Sci.*, **330**, 91.

STANTON, M.F., LAYARD, M. & TEGERIS, A. (1977). Carcinogenicity of fibrous glass: Pleural response in the rat in relation to fiber dimension. *J. Natl Cancer Inst.*, **58**, 587.

WAGNER, J.C., SLEGGS, C.A. & MARCHAND, P. (1960). Diffuse pleural mesothelioma and asbestos exposure in the Northwestern Cape Province. *Br. J. Int. Med.*, **17**, 260.

WAGNER, J.C. & BERRY, G. (1969). Mesothelioma in rats following inoculation with asbestos. *Br. J. Cancer*, **23**, 567.

WAGNER, J.C., BERRY, G. & TIMBRELL, V. (1983). Mesothelioma in rats after inoculation with asbestos and other materials. *Br. J. Cancer*, **28**, 193.


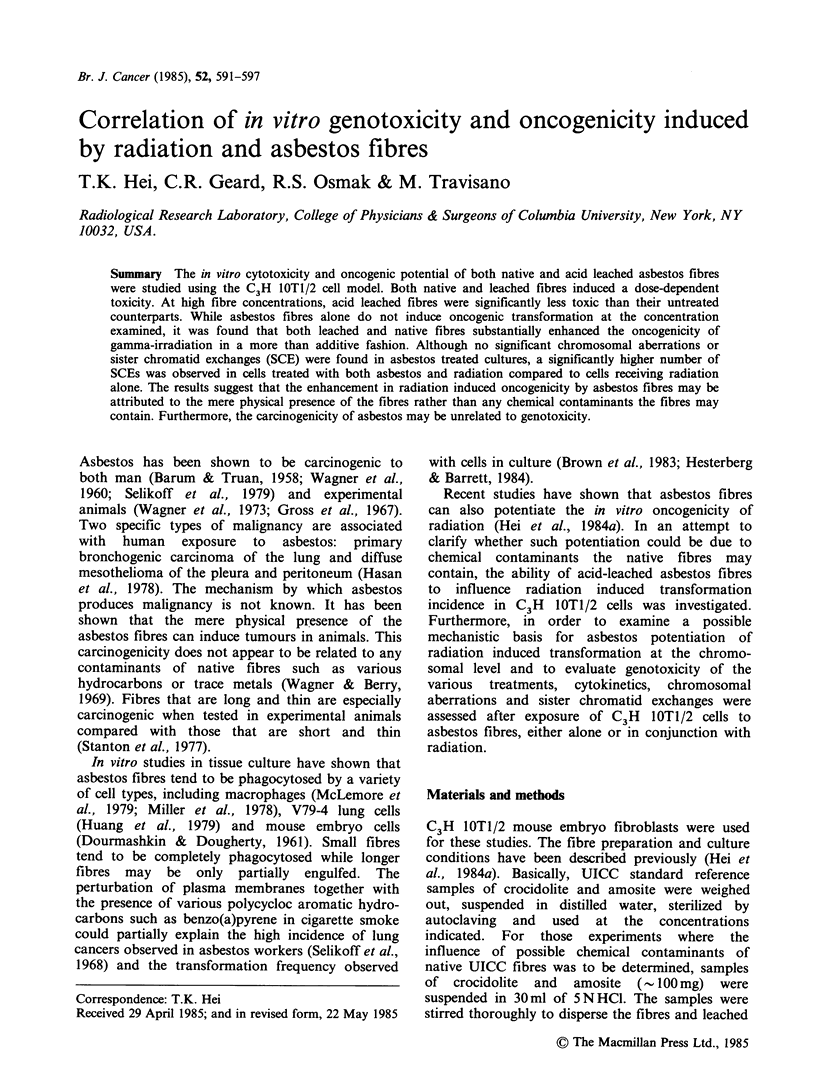

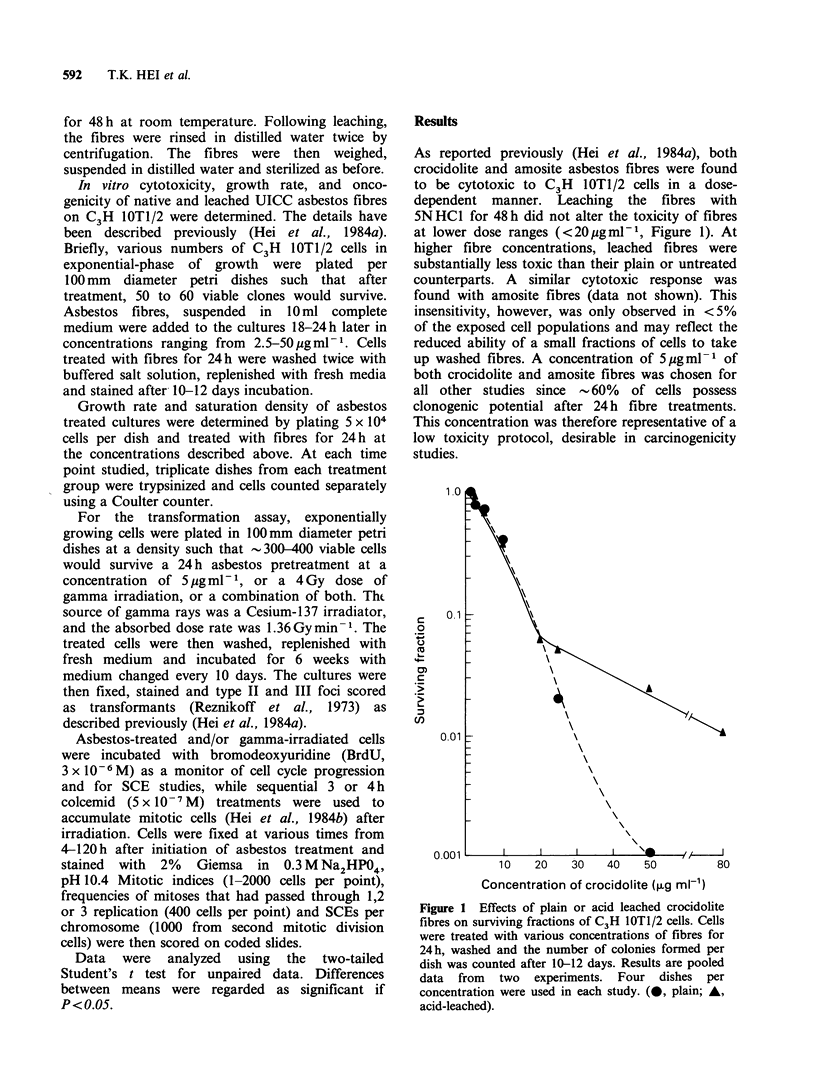

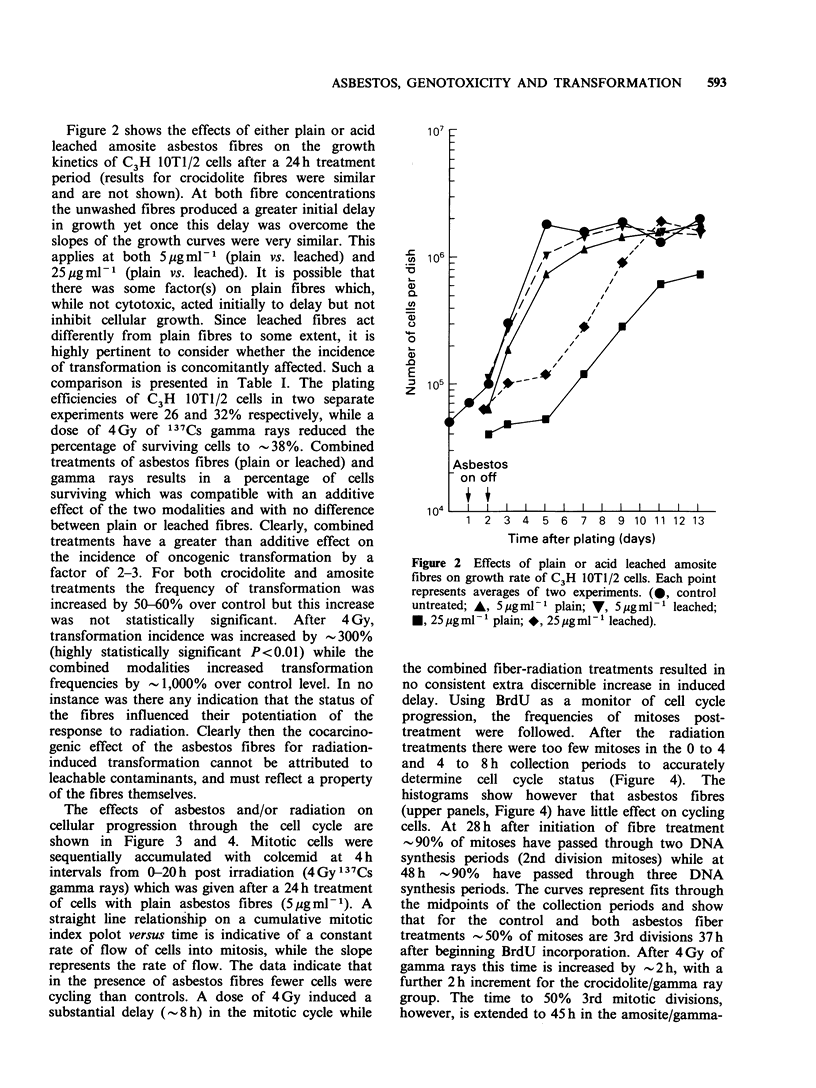

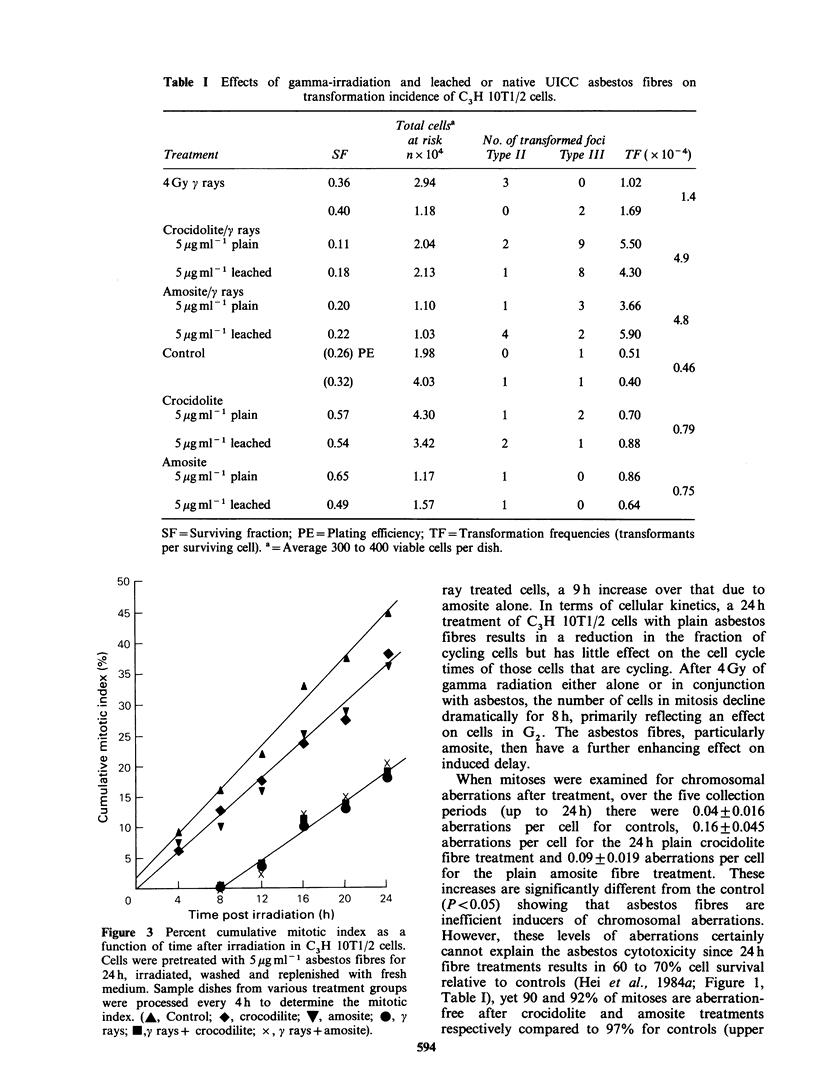

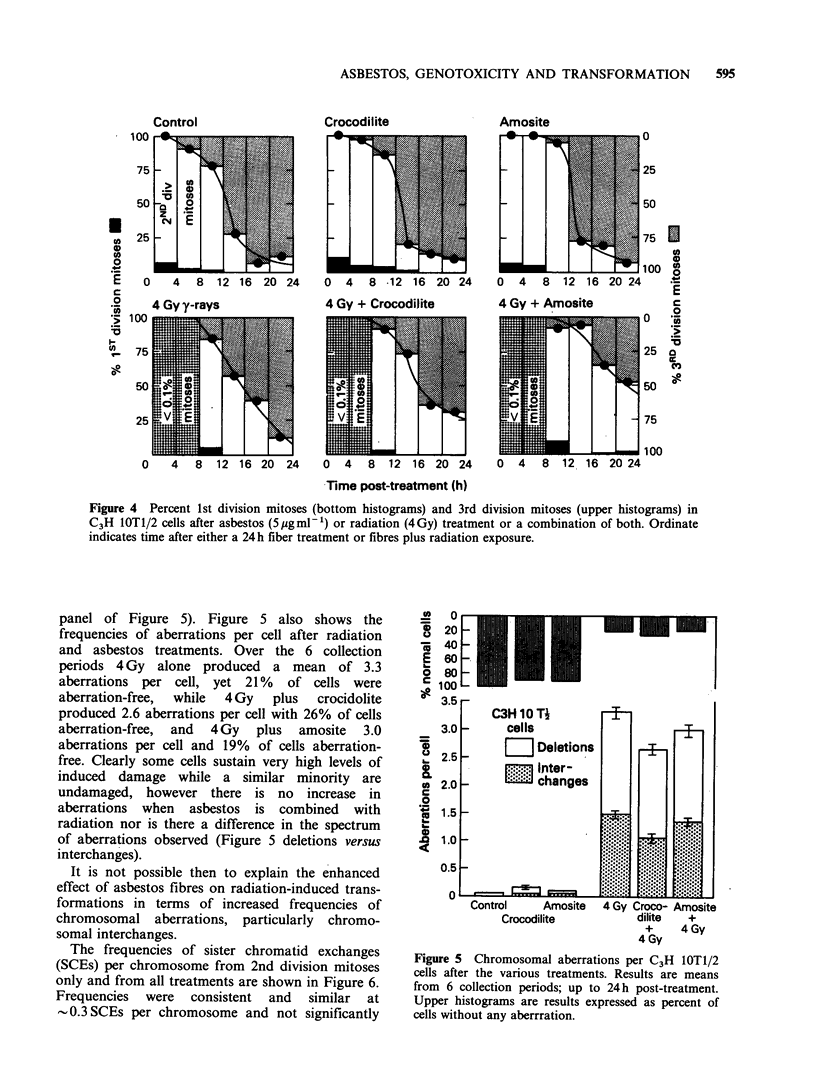

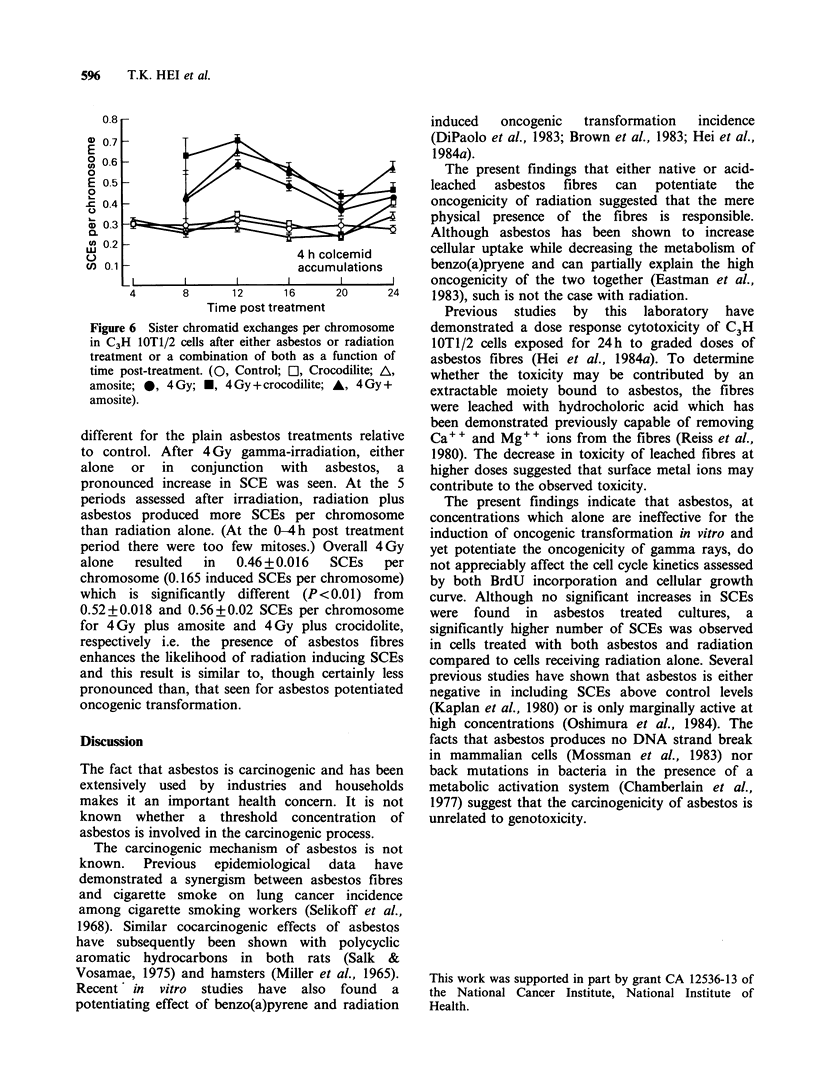

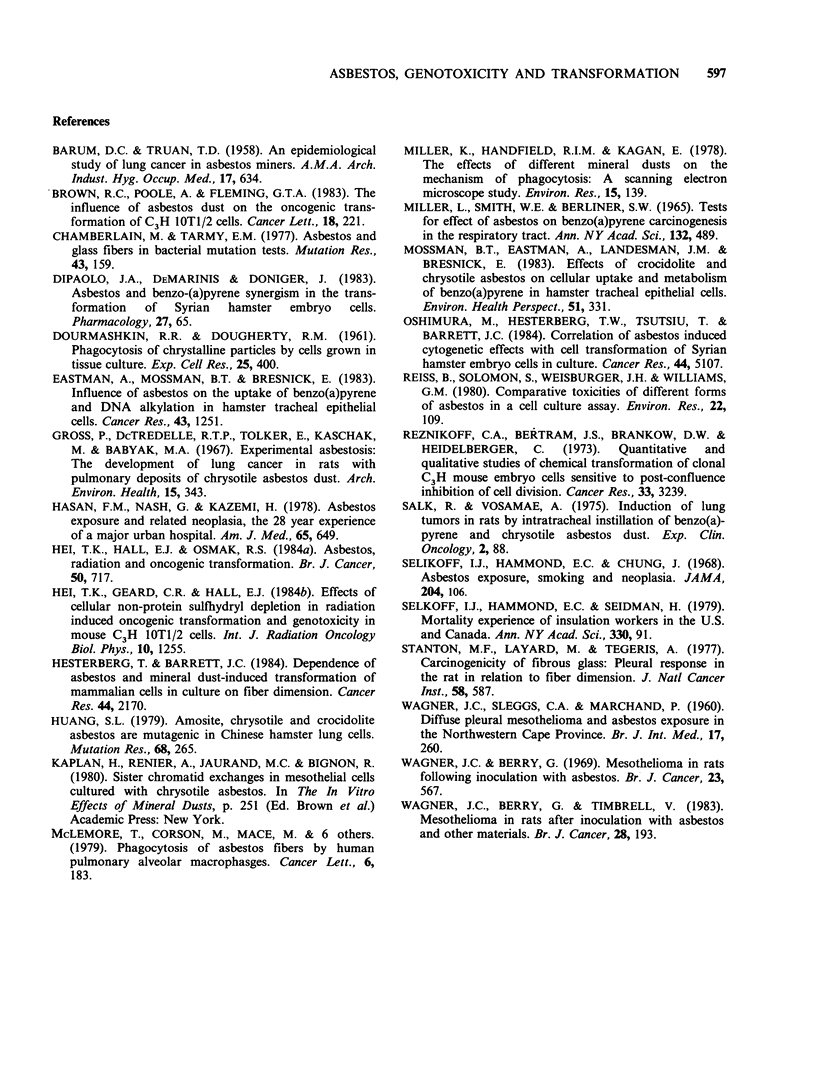


## References

[OCR_00717] Brown R. C., Poole A., Fleming G. T. (1983). The influence of asbestos dust on the oncogenic transformation of C3H10T 1/2 cells.. Cancer Lett.

[OCR_00722] Chamberlain M., Tarmy E. M. (1977). Asbestos and glass fibres in bacterial mutation tests.. Mutat Res.

[OCR_00738] Eastman A., Mossman B. T., Bresnick E. (1983). Influence of asbestos on the uptake of benzo(a)pyrene and DNA alkylation in hamster tracheal epithelial cells.. Cancer Res.

[OCR_00744] Gross P., DeTreville R. T., Tolker E. B., Kaschak M., Babyak M. A. (1967). Experimental asbestosis. The development of lung cancer in rats with pulmonary deposits of chrysotile asbestos dust.. Arch Environ Health.

[OCR_00751] Hasan F. M., Nash G., Kazemi H. (1978). Asbestos exposure and related neoplasia. The 28 year experience of a major urban hospital.. Am J Med.

[OCR_00761] Hei T. K., Geard C. R., Hall E. J. (1984). Effects of cellular non-protein sulfhydryl depletion in radiation induced oncogenic transformation and genotoxicity in mouse C3H 10T1/2 cells.. Int J Radiat Oncol Biol Phys.

[OCR_00756] Hei T. K., Hall E. J., Osmak R. S. (1984). Asbestos, radiation and oncogenic transformation.. Br J Cancer.

[OCR_00768] Hesterberg T. W., Barrett J. C. (1984). Dependence of asbestos- and mineral dust-induced transformation of mammalian cells in culture on fiber dimension.. Cancer Res.

[OCR_00774] Huang S. L. (1979). Amosite, chrysotile and crocidolite asbestos are mutagenic in Chinese hamster lung cells.. Mutat Res.

[OCR_00786] McLemore T., Corson M., Mace M., Arnott M., Jenkins T., Snodgrass D., Martin R., Wray N., Brinkley B. R. (1979). Phagocytosis of asbestos fibers by human pulmonary alveolar macrophages.. Cancer Lett.

[OCR_00792] Miller K., Handfield R. I., Kagan E. (1978). The effect of different mineral dusts on the mechanism of phagocytosis: a scanning electron microscope study.. Environ Res.

[OCR_00798] Miller L., Smith W. E., Berliner S. W. (1965). Tests for effect of asbestos on benzo[a]pyrene carcinogenesis in the respiratory tract.. Ann N Y Acad Sci.

[OCR_00803] Mossman B. T., Eastman A., Landesman J. M., Bresnick E. (1983). Effects of crocidolite and chrysotile asbestos on cellular uptake and metabolism of benzo(a)pyrene in hamster tracheal epithelial cells.. Environ Health Perspect.

[OCR_00816] Reiss B., Solomon S., Weisburger J. H., Williams G. M. (1980). Comparative toxicities of different forms of asbestos in a cell culture assay.. Environ Res.

[OCR_00822] Reznikoff C. A., Bertram J. S., Brankow D. W., Heidelberger C. (1973). Quantitative and qualitative studies of chemical transformation of cloned C3H mouse embryo cells sensitive to postconfluence inhibition of cell division.. Cancer Res.

[OCR_00835] Selikoff I. J., Hammond E. C., Churg J. (1968). Asbestos exposure, smoking, and neoplasia.. JAMA.

[OCR_00840] Selikoff I. J., Hammond E. C., Seidman H. (1979). Mortality experience of insulation workers in the United States and Canada, 1943--1976.. Ann N Y Acad Sci.

[OCR_00845] Stanton M. F., Laynard M., Tegeris A., Miller E., May M., Kent E. (1977). Carcinogenicity of fibrous glass: pleural response in the rat in relation to fiber dimension.. J Natl Cancer Inst.

[OCR_00851] WAGNER J. C., SLEGGS C. A., MARCHAND P. (1960). Diffuse pleural mesothelioma and asbestos exposure in the North Western Cape Province.. Br J Ind Med.

[OCR_00857] Wagner J. C., Berry G. (1969). Mesotheliomas in rats following inoculation with asbestos.. Br J Cancer.

